# Fast and Low-Cost Surface-Enhanced Raman Scattering (SERS) Method for On-Site Detection of Flumetsulam in Wheat

**DOI:** 10.3390/molecules25204662

**Published:** 2020-10-13

**Authors:** Mingming Han, Hongmei Lu, Zhimin Zhang

**Affiliations:** College of Chemistry and Chemical Engineering, Central South University, Changsha 410083, China; mingminghan@csu.edu.cn (M.H.); hongmeilu@csu.edu.cn (H.L.)

**Keywords:** SERS, flumetsulam, AuNPs, wheat

## Abstract

The pesticide residues in agri-foods are threatening people’s health. This study aims to establish a fast and low-cost surface-enhanced Raman scattering (SERS) method for the on-site detection of flumetsulam in wheat. The two-step modified concentrated gold nanoparticles (AuNPs) acted as the SERS substrate with the aid of NaCl and MgSO_4_. NaCl is served as the activator to modify AuNPs, while MgSO_4_ is served as the aggregating agent to form high-density hot spots. The activation and aggregation are two essential collaborative procedures to generate remarkable SERS enhancement and achieve the trace-level detection of flumetsulam. This method exhibits good enhancement effect with an enhancement factor of 10^6^ and wide linear range (5–1000 μg/L). With simple pretreatment, the flumetsulam residue in real wheat samples can be successfully detected with the limit of detection (LOD) down to 0.01 μg/g, which is below the maximum residue limit of flumetsulam in wheat (0.05 μg/g) set in China. The recovery of flumetsulam residue in wheat ranges from 88.3% to 95.6%. These results demonstrate that the proposed SERS method is a powerful technique for the detection of flumetsulam in wheat, which implies the great application potential in the rapid detection of other pesticide residues in various agri-foods.

## 1. Introduction

Flumetsulam is a member of the triazolopyrimidine sulfonanilide family herbicides. It was one of the most popular herbicides for soybeans, corn, wheat, barley, clover, alfalfa, peas, and other crops globally due to its good crop selectivity, broad spectrum activity on many broad-leaf weeds, low use rates, and low toxicity to mammals [1,2,3,4,5]. It has been used as a herbicide for post-emergence control since 1990 [6]. Flumetsulam can inhibit the enzyme acetolactate synthase, which is essential for the synthesis of branched chain amino acid (valine, leucine, and isoleucine) in weed growth [4,7,8]. Although flumetsulam is a herbicide with low acute toxicity, its residual period is relatively long, and the safety risks cannot be ignored because of large-scale or irregular usage [9,10]. To alleviate these problems, the Ministry of Agriculture of China has limited the maximum residue limits (MRL) of flumetsulam in wheat and corn to 0.05 mg/kg (GB 2763-2019). With the widespread application of pesticides in modern agriculture, their residues in water, soil, and crops have posed a great threat to the environment, humans, and animals. Therefore, the fast and low-cost detection of flumetsulam residue in agri-foods would be a feasible and necessary approach to ensure food safety.

Traditional analytical techniques have been widely applied to detect the pesticide residues in agri-foods, including gas chromatography (GC) [11], high-performance liquid chromatography (HPLC) [12,13], gas chromatography-mass spectrometry (GC-MS) [14,15,16], and liquid chromatography-mass spectrometry (LC-MS) [17,18,19]. These analytical techniques are accurate and capable of quantifying pesticide residues in a complex food matrix. However, they are time-consuming and often require complex sample pretreatment. Furthermore, the expensive instruments and complicated operations make the on-site analysis of pesticide residues in a complex food matrix difficult. Therefore, a simple and low-cost method employing the portable equipment is in urgent need for the rapid and accurate quantitation of flumetsulam residue in agri-foods.

Surface-enhanced Raman scattering (SERS) is a vibrational spectroscopy technique for rapidly identifying, characterizing, and detecting numerous biological and chemical targets [20,21,22,23]. It is an effective combination of Raman spectroscopy and nanotechnology, which not only retains the advantages of the molecular fingerprint specificity and narrow spectral bandwidth of Raman spectroscopy but also greatly enhances the weak Raman scattering signal with the assistance of noble metal nanoparticles [24,25,26,27]. Generally, there are two widely acknowledged mechanisms for the SERS enhancement, namely electromagnetic enhancement (EM) and chemical enhancement (CE) mechanisms [28,29,30,31]. The CE mechanism is also known as the charge transfer (CT) mechanism, which is based on the electronic interaction between the metal surface and adsorbed molecules [32]. The chemical enhancement is a short-range effect, and it usually only takes effect on the first layer of molecules adsorbed on the atomically rough metal surface. In addition, the CE mechanism may involve the formation of a metal–molecule electronic transition complex, where molecules can be directly adsorbed to the metal surface or indirectly adsorbed with the aid of some electrolyte additives [33]. Compared with chemical enhancement range, the EM enhancement is a long-range effect. It attributes the enhancement to the enhanced local electric field near the metal surface, which is not related to the adsorbed species. The EM effect is paramount when investigating substrates of SERS, such as the “hot spot” effect of the nanoscale rough surface of noble metals [34,35,36,37,38]. With the aid of the CE effect and EM effect, SERS can enhance the weak inelastic Raman scattering signal tremendously. In short, SERS has many unique detection advantages, such as low-cost, easy sample preparation, rapid signal readout, high sensitivity, non-destructive data collection, and detailed molecular fingerprint information [39]. These advantages make SERS a promising tool for the qualitative identification and quantitative analysis of flumetsulam residues in agri-foods.

**Scheme 1:**
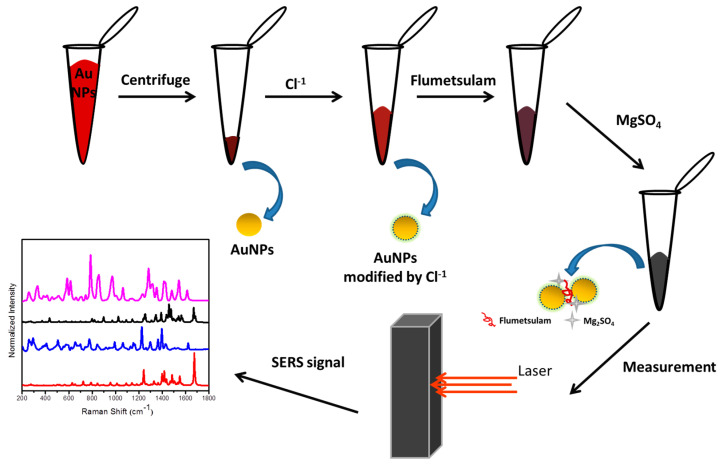
Schematic diagram of fabrication of the two-step modified concentrated AuNPs substrate in the detection of flumetsulam residue.

In this work, we aimed to explore the application of SERS approach to detect flumetsulam residue in agri-foods by using commercial wheat as an example. The halide-modified concentrated AuNPs were used as the activated SERS substrate for the detection of flumetsulam residue with the aid of a sulfate aggregating agent. An extremely simple method was used to extract the flumetsulam residue in real wheat samples, and AuNPs with optimal particle size and concentration were prepared; then, the surface of prepared AuNPs was modified with halide to obtain a highly effective SERS substrate. The schematic diagram of this method is depicted in Scheme 1. In addition, we also carried out experimental and theoretical studies on the Raman shift, characteristic Raman bands, and vibrational modes of flumetsulam with the assistance of density functional theory (DFT) calculation. The second derivative of SERS spectra was used to achieve better spectral resolution and quantification.

## 2. Results and Discussion

### 2.1. Characterization of AuNPs Substrates

**Figure 1:**
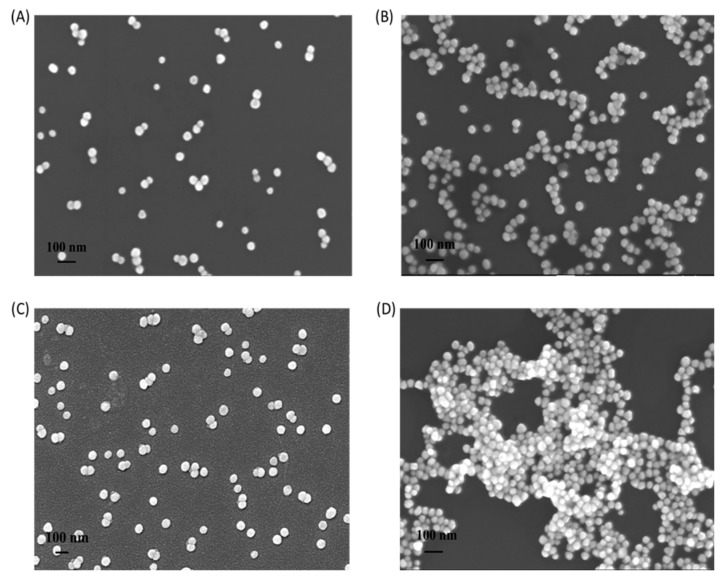
SEM images of different AuNPs (**A**): unconcentrated colloidal AuNPs; (**B**): 2× concentrated colloidal AuNPs; (**C**): NaCl-modified AuNPs; (**D**): NaCl-modified AuNPs with flumetsulam under the addition of MgSO_4_).

Figure 1 shows the SEM images of AuNPs in different samples. It can be observed from Figure 1A,B that the colloidal AuNPs are in spherical shape and dispersed homogeneously, with an average diameter of about 45 nm. Figure 1A,B show the SEM images of the unconcentrated AuNPs and 2× concentrated AuNPs, respectively. It clearly shows that the concentrated AuNPs have denser and more homogeneous nanoparticles with a significantly reduced inter-particle gap but no aggregation or precipitation, indicating that the chance of hot spots generation is significantly increased. Moreover, it can be found that with the addition of NaCl (1 × 10^−3^ mol/L), the Cl-AuNPs (NaCl modified AuNPs) colloid does not produce visible aggregation (Figure 1C), but there is a slight aggregation appearing when the flumetsulam is added into the Cl-AuNPs colloid (Figure S1). However, it is clearly shown that after the addition of MgSO_4_, Cl-AuNPs with flumetsulam display an apparent aggregation (Figure 1D), implying that the MgSO_4_ may act as an aggregating agent.

**Figure 2:**
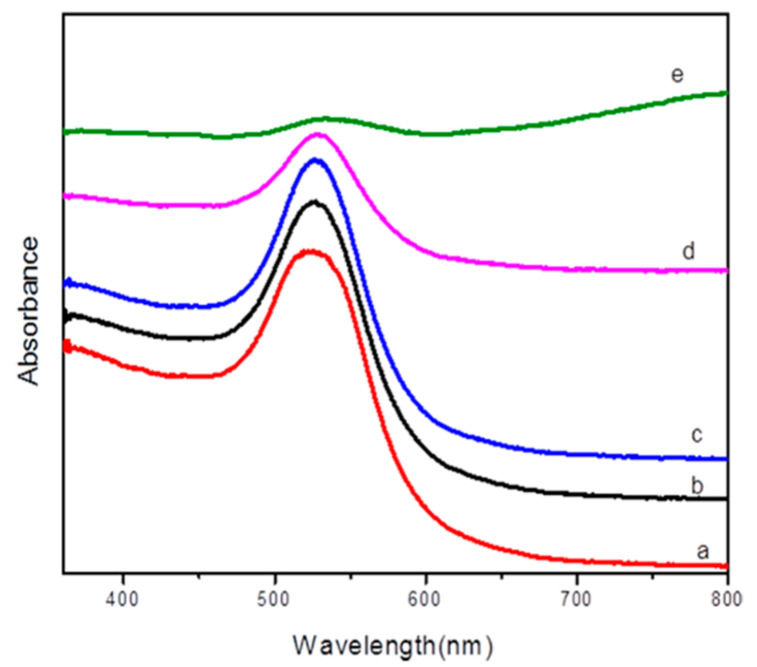
UV-vis absorption spectra of five different samples (a: AuNPs colloid, b: 2× concentrated AuNPs colloid, c: NaCl-modified AuNPs colloid, d: NaCl-modified AuNPs colloid with flumetsulam (1000 μg/L), and e: NaCl-modified AuNPs colloid with flumetsulam (1000 μg/L) under the addition of MgSO_4_).

Figure 2 shows the UV-vis absorption spectra of different AuNPs colloid samples. For the Au colloid, 2× concentrated AuNPs colloid, and Cl-AuNPs colloid, their maximum absorbance peaks all appear at 525 nm (curves a, b and c), which can be attributed to the localized surface plasmon resonance (LSPR) absorption of AuNPs [40]. The concentration (2×) and modification by NaCl (1 × 10^−3^ mol/L) of AuNPs colloid hardly cause the shift of the maximum LSPR adsorption band, indicating that there is no visible aggregation in the 2× concentrated AuNPs colloid and Cl-AuNPs colloid. The narrow UV-Vis absorption band of AuNPs colloid indicates that AuNPs are monodispersed without aggregation or precipitation [20]. By contrast, when the Cl-AuNPs colloid is mixed with the flumetsulam solution, the LSPR maximum absorption is slightly red shifted to 528 nm (curve d), which should be the result of the interaction between Cl-AuNPs and flumetsulam molecules. When MgSO_4_ aqueous solution is added into the above mixture, the LSPR maximum absorption is red shifted to 533 nm (curve e). In addition, it is very interesting that the originally narrow UV-Vis absorption band becomes significantly wider and weaker. These changes in curve e can be attributed to the fact that there is an obvious aggregation of AuNPs caused by MgSO_4_ aggregating agent, which is exactly consistent with the measurement results of SEM.

To evaluate the enhancement performance of the two-step modified concentrated AuNPs substrate (NaCl activator-modified AuNPs with an additional MgSO_4_ aggregating agent added), the characteristic Raman band intensity at 1510 cm^−1^ of the typical probe molecule R6G was selected to calculate the analytical enhancement factor (AEF) according to Formula (1) [41]:

(1) $$AEF=\frac{I_{SERS}}{I_{normal}}\times \frac{C_{normal}}{C_{SERS}}.$$

Here, *I_SERS_* and *I_normal_* are intensities of the characteristic Raman band at 1510 cm^−1^ of R6G obtained by SERS and the normal Raman, respectively. *C_SERS_* and *C_normal_* are the molar concentrations of R6G in the SERS and normal Raman, respectively. The AEF of the proposed SERS method could reach 7.67 × 10^6^. The limit of detection (LOD) of R6G calculated by Formula (2) [42] can be as low as about 10^−9^ M. The *S_b_* represents the standard deviation of the response of the blank samples, and *b* represents the slope of the calibration curve.

(2) $$LOD=\frac{3S_{b}}{b}$$

### 2.2. Experimental and Theoretical Raman Spectra of Flumetsulam

**Figure 3:**
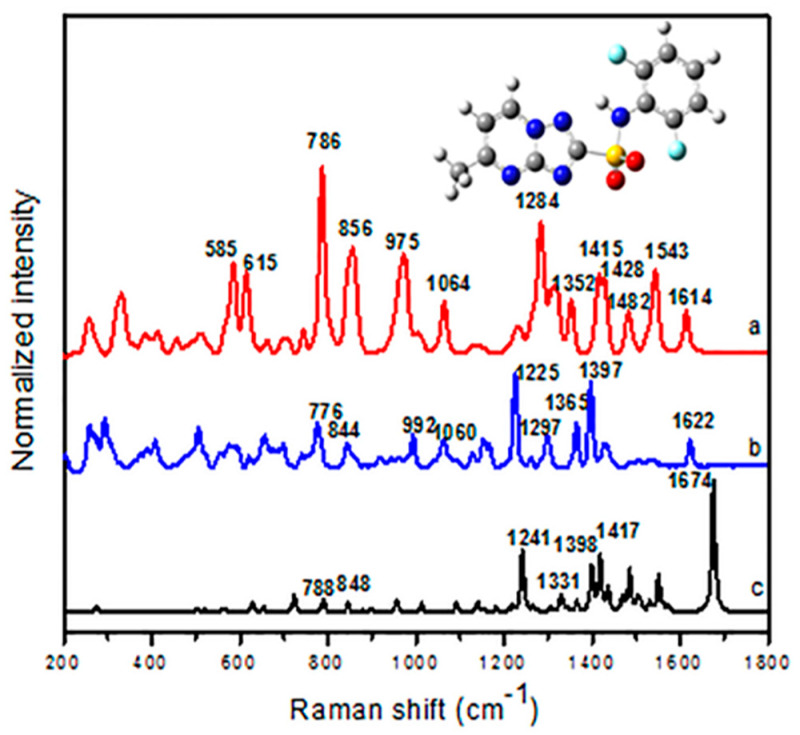
Theoretical and experimental spectra of the flumetsulam molecule (a: Experimental surface-enhanced Raman scattering (SERS) spectrum, b: Experimental normal Raman spectrum of flumetsulam powder, c: Simulated Raman Spectra of the free flumetsulam molecule).

To better understand the characteristic peaks and vibrational modes of flumetsulam, the theoretical normal Raman spectrum of the free flumetsulam molecule was obtained through DFT calculation (Figure 3). Comparing the experimental spectrum with the theoretical spectrum, the corresponding vibration modes of the main peaks are listed in Table S1. However, the SERS spectrum (using the two-step modified concentrated AuNPs substrate) of flumetsulam exhibited obvious differences near 585 to 856 cm^−1^ with the theoretical and experimental normal Raman spectrum of the free flumetsulam molecule, which are attributed to different types of pyrimidine ring, triazole ring bending, and C-S stretching. The changes in the Raman spectrum of flumetsulam originate from the interaction between the flumetsulam molecule and surface of the two-step modified concentrated AuNPs. Compared with the normal Raman spectrum of flumetsulam powder (Figure 3b), some bands of the flumetsulam solution shift or split in the SERS spectrum (Figure 3a), which is accompanied by the appearance of some new bands or the disappearance of the original bands. For instance, the band at 1397 cm^−1^ in the normal Raman spectrum splits into two peaks at 1415 and 1428 cm^−1^ in the SERS spectrum. These bands at 776 and 844 cm^−1^, which can be assigned to the stretching vibration of the pyrimidine ring and triazole ring in the normal Raman spectrum, shift to 786 and 856 cm^−1^ in the SERS spectrum, respectively. The band at 1622 cm^−1^ attributed to in-plane deformation vibration of the benzene ring in the normal Raman spectrum shifts to 1614 cm^−1^ in the SERS spectrum. In addition, two overlapping new bands appear at 585 and 615 cm^−1^ in the SERS spectrum of flumetsulam, which cannot be observed in the normal Raman spectrum. Furthermore, it is noteworthy that the relative intensities are significantly different between the normal Raman spectrum and the SERS spectrum. In the normal Raman spectrum, the strongest bands are located at 1225 and 1397 cm^−1^, while the strongest peak in the SERS spectrum is located at 786 cm^−1^. These differences between the normal Raman spectrum and the SERS spectrum of flumetsulam may be attributed to the nature of the adsorbate on the surface of AuNPs and the dual contribution of the NaCl and MgSO_4_ to the CE and EM effects [30,43].

### 2.3. The Influence of AuNPs Size and Concentration on SERS Detection of Flumetsulam

**Figure 4:**
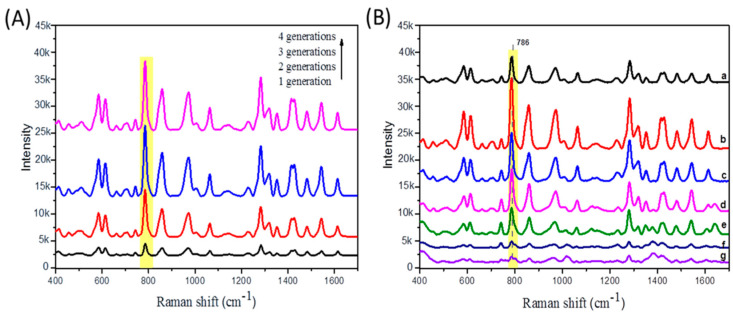
(**A**): SERS spectra of flumetsulam adsorbed on four different AuNPs substrates with different growth generations (1–4 generations). (**B**): SERS spectra of flumetsulam adsorbed on AuNPs substrates with different concentration factors (a: 1×, b: 2×, c: 5×, d: 10×, e: 20×, f: 40×, g: 100×).

To get a better SERS response of flumetsulam and deeper understanding on the enhancement mechanism, some explorations of the detection conditions were conducted. We first compared the SERS activity of AuNPs grown up to different generations (1, 2, 3, and 4 generations. One generation represents a growth process of the AuNPs size mentioned in Section 3.3). Figure 4A shows SERS spectra of flumetsulam adsorbed on four different AuNPs substrates with different growth generations. It can be found that under the same conditions, the characteristic Raman band intensity of flumetsulam always increases in the order of 1 generation < 2 generations < 4 generations ≤ 3 generations. The AuNPs grown up to 3 generations achieve the obvious SERS enhancement because of the AuNPs grown to this size are relatively stable, and flumetsulam can form the optimal coverage on the surface of AuNPs with this particle size. Therefore, the AuNPs grown up to 3 generations were selected as the initial substrate in follow-up experiments.

Figure 4B shows the SERS spectra of flumetsulam adsorbed on concentrated AuNPs substrates with different concentration factors. It can be seen that flumetsulam exhibits the strongest SERS enhancement on the 2× concentrated AuNPs substrate compared with other six concentrated AuNPs substrates (1×, 5×, 10×, 20×, 40×, and 100×). The spectrum of flumetsulam in unconcentrated AuNPs colloid (1×) shows weak Raman intensity. When the concentration factor is higher than 20×, the SERS signal of flumetsulam is weaker than the unconcentrated one. The SERS intensity of flumetsulam gradually decreases with increasing concentration factor (2–100×), and the SERS signal is almost invisible when it reaches 100×. Proper centrifugation can remove too small nanoparticles, which can improve both the stability and uniformity of the AuNPs colloid. Meanwhile, the plasmonic coupling between concentrated AuNPs in close proximity can dramatically enhance SERS signals of the analyte [44]. However, the excessive concentration factor may cause the aggregation or precipitation of AuNPs and weaken the SERS enhancement. Therefore, the 2× concentrated AuNPs substrate was fixed in the following experiments.

### 2.4. The Influence of Halide and Sulfate on SERS Detection of Flumetsulam

**Figure 5:**
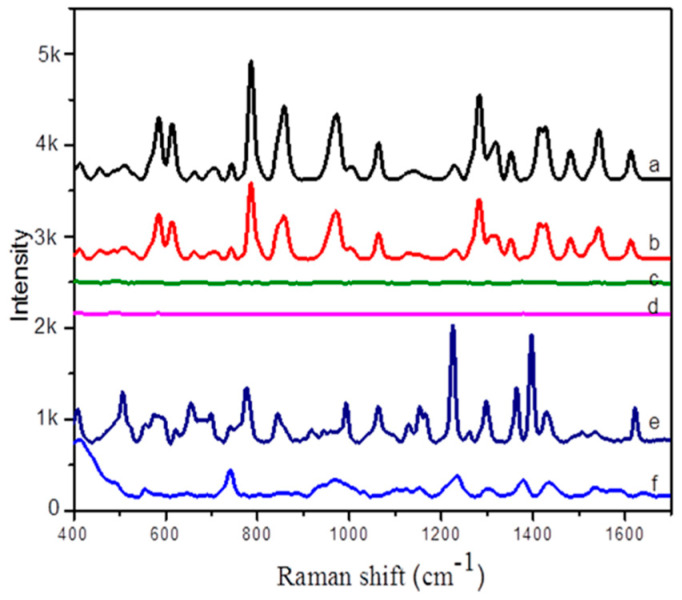
Spectra of flumetsulam (1000 μg/L) adsorbed on different AuNPs substrates (a: on the two-step modified concentrated AuNPs substrate. b: on AuNPs substrate with an additional MgSO_4_ added but without NaCl modification. c: on AuNPs substrate without any modification or extra addition. d: on AuNPs substrate with only NaCl modification). e: normal Raman spectrum of flumetsulam powder. f: blank solution on the two-step modified concentrated AuNPs substrate.

Under the above experimental conditions, the effects of halide and sulfate on SERS detection of flumetsulam were also investigated. Figure 5 shows SERS spectra of flumetsulam adsorbed on different AuNPs substrates, blank solution on the two-step modified concentrated AuNPs substrate, and the normal Raman spectrum of flumetsulam powder. The background noise of blank solution (curve f) was too weak to interfere with the detection of flumetsulam. Interestingly, flumetsulam adsorbed on the two-step modified concentrated AuNPs substrate (curve a) exhibits the maximal SERS enhancement. The flumetsulam adsorbed on AuNPs substrate with an additional MgSO_4_ added but without NaCl modification showed the second largest enhancement (curve b). The SERS signal of flumetsulam adsorbed on AuNPs substrate without any modification or extra addition (curve c) and flumetsulam adsorbed on AuNPs substrate with only NaCl modification (curve d) is almost invisible. This indicates that the modification of halide (NaCl) for AuNPs substrate and an extra MgSO_4_ addition are two essential collaborative procedures to obtain the significant SERS enhancement and achieve the successful detection of flumetsulam residue.

**Figure 6:**
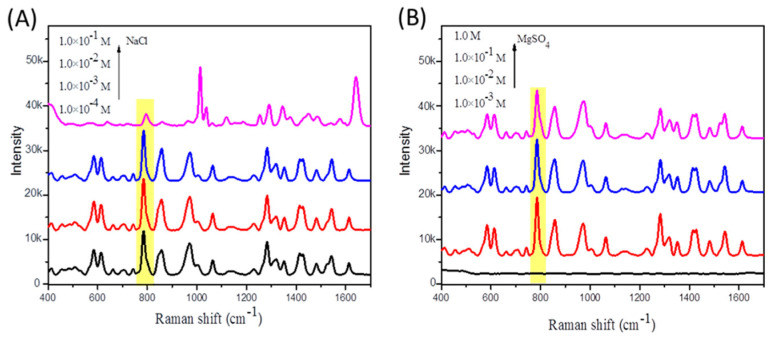
(**A**): SERS spectra of flumetsulam absorbed on the two-step modified concentrated AuNPs substrate with NaCl at different concentrations (1.0 × 10^−4^ to 1.0 × 10^−1^ mol/L). (**B**): SERS spectra of flumetsulam absorbed on the two-step modified concentrated AuNPs substrate with MgSO_4_ at different concentrations (1.0 × 10^−3^ to 1.0 mol/L).

In addition, it can be found from Figure 6A,B that the SERS activity of flumetsulam is obviously related to the concentrations of NaCl and MgSO_4_. The maximal SERS enhancement of flumetsulam can been achieved when the concentration of NaCl and MgSO_4_ are 1 × 10^−3^ and 1 × 10^−2^ mol/L, respectively. These concentrations were fixed in the following experiments.

Currently, there are three mechanisms to explain the effect of adding salts to the noble metal colloid: activation, aggregation, and desorption [28,43,45]. Activation usually occurs in low concentrations of halides, where metal nanoparticles do not aggregate, but their SERS activity will be immediately enhanced. The halides can form SERS-active sites on the metal nanoparticles surface, inducing intrinsic electronic interaction between the adsorbate and the nanoscale metals [46]. Aggregation is usually experienced at a relatively high halide concentration, where the local electric field near the metal surface will increase, generating hot spots. Desorption is the removal of adsorbates from metal nanoparticles. One can observe from the characterization analysis of SEM and UV-vis absorption spectra that the addition of NaCl at a low concentration (1 × 10^−3^ mol/L) hardly results in any obvious aggregation. It indicates that the mechanism of the enhancement caused by the modification of AuNPs by NaCl may be the “activation effect” rather than “aggregation effect”. When adding more NaCl to the Au colloid, the SERS activity of flumetsulam drops, which may relate to the coverage of the SERS-active sites by NaCl rather than flumetsulam (desorption). According to Figure 5, the intensities of Raman bands were significantly different for the two-step modified concentrated AuNPs substrate (curve a) and the substrate with an additional MgSO_4_ added but without NaCl modification (curve b). This also indicates that SERS-active sites of AuNPs can be activated by NaCl, and NaCl works as an activator for the formation of the Au–flumetsulam electronic transition complex [47]. The above results suggest that flumetsulam molecules may be chemisorbed on the AuNPs surface in the presence of NaCl, and the CE effect should be the main mechanism responsible for this remarkable enhancement [48]. However, it can be seen from Figure 5 that the SERS signal of flumetsulam adsorbed on the concentrated AuNPs substrate with only NaCl modification (curve d) is almost invisible, but there is a surprising enhancement after a slight amount of addition of MgSO_4_ (curve a). This indicates that the SERS-active sites on relatively isolated AuNPs are not sufficient to generate obvious SERS enhancement, even after activation by NaCl. Accordingly, the intense local electric field of “hot spots” between two or multiple touching nanoparticles is crucial to magnify Raman signals in SERS, that is, the aggregation effect of MgSO_4_. MgSO_4_ plays the role of aggregating agent in this detection system, and its concentration has a crucial effect on SERS detection of flumetsulam. MgSO_4_ at appropriate concentrations can aggregate AuNPs to form more “hot spots” and enhance the condensed state of the Au–flumetsulam complex, thereby enhancing the SERS signal. However, excessive MgSO_4_ may cause the irreversible flocculation of AuNPs, lead to the destabilization and collapse of colloidal particles, and weaken the SERS enhancement. All the above results confirm that the activation by NaCl and aggregation by MgSO_4_ are two essential collaborative procedures for the successful SERS detection of flumetsulam.

**Figure 7:**
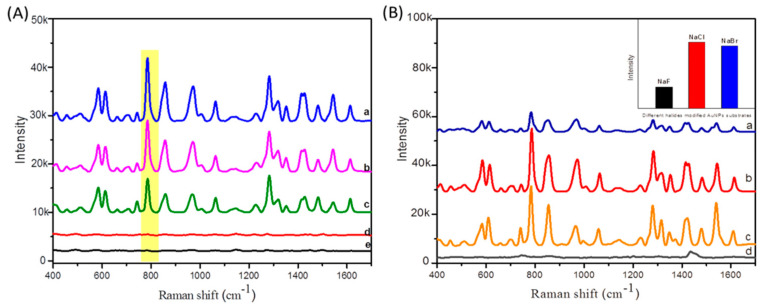
(**A**): SERS spectra of flumetsulam absorbed on Cl-AuNPs substrates with the addition of different sulfates (a: MgSO_4_, b: ZnSO_4_, c: Al_2_(SO_4_)_3_, d: K_2_SO_4_, and e: (NH_4_)_2_SO_4_), respectively. (**B**): SERS spectra of flumetsulam absorbed on different halides-modified AuNPs substrates (a, b, c, and d represent NaF, NaCl, NaBr, and NaI respectively).

To further investigate the effect of sulfate aggregating agents with different cations on the SERS performance, the SERS spectra of flumetsulam on the Cl-AuNPs substrate with different aggregating agents ((NH_4_)_2_SO_4_, K_2_SO_4_, MgSO_4_, ZnSO_4_ and Al_2_(SO_4_)_3_) were collected. These spectra are shown in Figure 7A. It can be seen that the intensities of the characteristic Raman bands of flumetsulam always increase in the order of (NH_4_)_2_SO_4_, K_2_SO_4_ < Al_2_(SO_4_)_3_ < ZnSO_4_ ≤ MgSO_4_. Flumetsulam on the Cl-AuNPs substrate with the addition of MgSO_4_ exhibits the maximal SERS enhancement under the same condition, which may be caused by the difference of the cationic charge in sulfates [49]. Compared with NH_4_^+^, K^+^, and Al^3+^, Mg^2+^ and Zn^2+^ have two positive charges and show better SERS enhancement effects. It may be because insufficient (NH_4_^+^ and K^+^) or excessive (Al^3+^) positive charges of cations are not conducive to the formation of efficient “hot spots” and enhancement of the condensed state of the AuNPs–flumetsulam complex. When ZnSO_4_ was used as the aggregating agent, the characteristic Raman band intensity of flumetsulam is slightly weaker than that of MgSO_4_ (curves a and b), which may be attributed to the different electronegativity between Mg^2+^ and Zn^2+^ [50]. The electronegativity of Mg^2+^ (17.13) is obviously higher than that of Zn^2+^ (10.38), and stronger electrostatic interaction allows AuNPs to aggregate more efficiently and form more hot spots.

Furthermore, we compared the SERS activity of four different halide-modified AuNPs substrates (NaF, NaCl, NaBr, and NaI). As can be seen from Figure S3, the best SERS enhancement performance of flumetsulam can been achieved when the concentration of these halides is all at 1 × 10^−3^ mol/L (AuNPs modified by NaI at these different concentrations had no SERS activity). In addition, it can be seen from Figure 7B that among the four kinds of halides (under the optimal concentration conditions), the characteristic Raman band intensity at 786 cm^−1^ of flumetsulam always decreases in the order of NaCl > NaBr > NaF > NaI. The modifications of AuNPs substrates by NaF, NaCl, and NaBr can enhance the SERS signal of flumetsulam. However, the SERS signal of flumetsulam adsorbed on the AuNPs substrate with NaI modification cannot be observed at all. These four halide ions have different electronegativity and ionic radius, which may lead to different degrees of competitive adsorption on the AuNPs surface, different degree modifications of AuNPs, and different activation effects [49]. The electronegativity of I^−^ is much smaller than that of F^−^, Cl^−^, and Br^−^, and its competitive adsorption on the surface of AuNPs is extremely weak [51]. Therefore, its surface modification effect on AuNPs is not as effective as that of F^−^, Cl^−^, and Br^−^, resulting in low SERS activity. However, due to the high electronegativity of F^−^, its competitive adsorption effect on the AuNPs surface is too strong, which may weaken the adsorption of flumetsulam on the active regions of the AuNPs surface, resulting in lower SERS performance than Cl^−^ and Br^−^. The electronegativity of Cl^−^ and Br^−^ is similar, so the SERS enhancement caused by them is also very similar. However, the SERS activity caused by the modification of Cl^−^ for AuNPs substrates is slightly stronger than that of Br^−^. The subtle difference is mainly due to the fact that Cl^−^ and Br^−^ can give rise to different degrees of surface modifications on AuNPs and lead to different degrees of activation effects for AuNPs because of their different electronegativity. Obviously, Cl^−^ should be more suitable for the surface modification and activation of AuNPs. In addition, from the perspective of steric hindrance, the ion radius of F^−^, Cl^−^, Br^−^, and I^−^ are in the order of F^−^ (136 pm) < Cl^−^ (181 pm) < Br^−^ (196 pm) < I^−^ (216 pm), respectively [52]. The steric hindrance of I^−^ between flumetsulam molecules and AuNPs surface is obviously greater. Therefore, 1 × 10^−3^ mol/L of NaCl was selected to modify AuNPs substrates in subsequent experiments.

### 2.5. Quantitative Analysis of Flumetsulam

**Figure 8:**
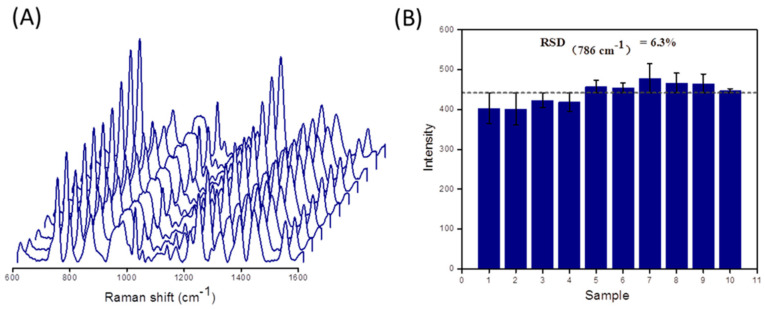
(**A**) Ten random SERS spectra of flumetsulam absorbed on the two-step modified concentrated AuNPs substrate under the same conditions; (**B**) The histogram of Raman intensity for each random SERS measurement (the error bar indicates the signal derivation of ten random SERS measurements from different samples).

The stability and reproducibility of the developed SERS method was evaluated before quantitative analysis. It can be seen from Figure 8 that the main characteristic band intensities of flumetsulam by ten random measurements are almost the same. Taking the peak intensity of 786 cm^−1^ as the evaluation standard, the relative standard deviation (RSD) is calculated as 6.3%, verifying that the SERS method is reliable for the detection of flumetsulam. In addition, the AEF of flumetsulam was calculated to be 5.29 × 10^6^ according to Formula (1). Results indicated that this two-step modified concentrated AuNPs substrate has good SERS activity and repeatability. Therefore, it was used for the quantitative analysis of flumetsulam.

**Figure 9:**
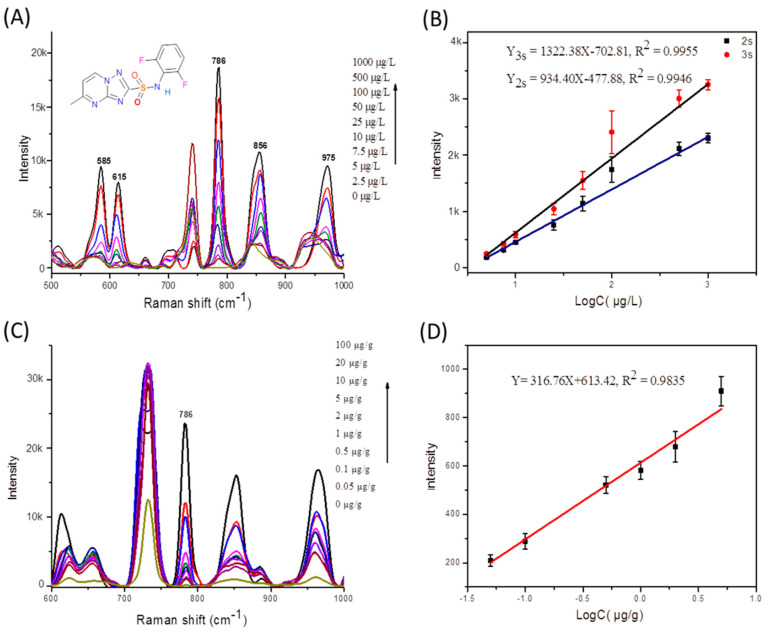
(**A**): SERS spectra of flumetsulam with different concentrations absorbed on the two-step modified concentrated AuNPs substrate. (**B**): The plot of the intensity of SERS peak at 786 cm^−1^ versus the logarithm of flumetsulam concentration (5–1000 μg/L. The error bars indicate standard deviations; 2 s and 3 s are the collecting time of SERS spectra). (**C**): The spectra of flumetsulam residue from extraction solutions of different concentrations spiked wheat samples with the proposed SERS method. (**D**): The plot of the intensity of the SERS peak at 786 cm^−1^ versus the logarithm of flumetsulam concentration in wheat (0.05–5 μg/g. The error bars indicate standard deviations).

A series of SERS spectra of flumetsulam solution with different concentrations were acquired under above optimized conditions (Figure 9A). Encouragingly, the Raman band at 786 cm^−1^ attributed to the characteristic vibration of the pyrimidine ring can still be observed on the two-step modified concentrated AuNPs substrate when the concentration of flumetsulam decreases to 2.5 μg/L. However, other Raman bands almost cannot be identified because they are either too weak or poorly shaped. Therefore, the SERS intensity of the characteristic peak at 786 cm^−1^ was selected to quantify the flumetsulam residue level. Figure S4 exhibits the second derivative SERS spectra of flumetsulam with different concentrations, which clearly reveals the spectral features of flumetsulam vibrational modes in the Raman shift range of 580–850 cm^−1^. Moreover, a good linear relationship spans the wide concentrations from 5 to 1000 μg/L, as plotted by SERS intensity at 786 cm^−1^ and the logarithm of flumetsulam concentration. The linear equation can be described as Y = 934.40X − 477.88 with an R^2^ of 0.9946 (Figure 9B). The limit of detection (LOD) is calculated to be 1.92 μg/L. It demonstrates that this SERS method is an effective and reliable method for the quantitative analysis of flumetsulam.

### 2.6. Detection of Flumetsulam Residue in Real Wheat Samples

Flumetsulam residue in wheat was analyzed under the optimized conditions to verify the feasibility of this SERS method in real samples. First, blank wheat samples were spiked with flumetsulam to obtain samples with different residue levels of 100, 50, 10, 5, 2, 1, 0.5, 0.2, 0.1, and 0 μg/g. Then, these spiked wheat samples were extracted using the pretreatment method mentioned in Section 3.5, and the final solution was directly used for SERS detection. Figure 9C shows the spectra of the wheat extraction solution containing flumetsulam. The prominent characteristic Raman band at 786 cm^−1^ can still be observed in the SERS spectra of wheat samples spiked with flumetsulam at the level of 0.05 μg/g, and the LOD could be as low as 0.01 μg/g, which matched the maximum residue limit of flumetsulam in wheat (0.05 μg/g) set in China (GB 2763-2019). The spike-and-recovery test was carried out by spiking the wheat samples at three levels, and the results are listed in Table 1. It shows that the recoveries of flumetsulam were in the range of 88.3–95.6% with RSDs between 5.6% and 8.9%. All these results reveal that the proposed SERS method is reliable for the quantitative detection of flumetsulam residue in the real wheat samples.

Compared with other analytical methods of flumetsulam residue reported in the literature [1,4,5,6], the sample preparation and the SERS detection procedure of this method is extremely simple, does not require tedious sample extraction and additional purification procedures, and does not require multiple expensive reagents. Furthermore, the SERS technology also has the unique advantages of rapid signal readout and non-destructive data collection. As the result, the easy, rapid, and on-site analysis of flumetsulam residue can be achieved with the developed SERS method and a portable Raman spectrometer.

## 3. Materials and Methods

### 3.1. Materials and Reagents

Gold chloride trihydrate (HAuCl_4_·3H_2_O) and sodium citrate dihydrate (Na_3_C_6_H_5_O_7_·2H_2_O) were purchased from Sigma-Aldrich (Shanghai, China). Pure flumetsulam (98%) was purchased from Aladdin Reagent Co. Ltd. (Shanghai, China). Rhodamine 6G (R6G) was purchased from Xiya Chemical Industry Co. Ltd. (Shandong, China). Acetone was purchased from Chengdu Kelong Chemical Preparation Co. Ltd. (Chengdu, China). Hexane was purchased from Shanghai Titanchem Co. Ltd. (Shanghai, China). Sodium fluoride, sodium chloride, sodium bromide, sodium iodide, ammonium sulfate, potassium sulfate, magnesium sulfate, zinc sulfate, and aluminum sulfate were purchased from Shanghai Titan Scientific Co. Ltd. (Shanghai, China). Wheat samples were purchased from a local supermarket (Changsha, China). All of these reagents were used without further purification. All glassware was cleaned with aqua regia before usage. Ultrapure water (18.2 MΩ) was used throughout the study.

### 3.2. Apparatus

The surface morphology and size of the prepared AuNPs were characterized by a field emission scanning electron microscope (JSM-7610FPlus, JEOL Ltd., Tokyo, Japan). Ultraviolet-visible (UV-Vis) absorption spectra were obtained by a UV-Vis spectrometer (UV-2450, Shimadzu Suzhou Instruments Co. Ltd., Kyoto, Japan). SERS spectra were recorded by a portable Raman spectrometer (i-Raman Pro, B&W Tek Inc., Newark, DE, USA). The 785 nm radiation was used as excitation source (output laser power ≈390 mW). The typical collecting time was 2 s for each Raman spectrum with one time accumulation, and the laser was set at 30% power (≈117 mW) unless otherwise stated. The measurements were repeated at least three times for each sample from different locations.

### 3.3. Preparation of AuNPs with Different Sizes

The AuNPs were synthesized using a seed-mediated growth method based on a previous study [53]. Briefly, 150 mL of sodium citrate (2.2 mM) aqueous solution was added into a 250 mL three-necked round-bottomed flask equipped with a condenser to prevent the solvent evaporation. It was heated to 100 °C and kept for 15 min under vigorous stirring. Then, 1 mL of HAuCl_4_ (25 mM) was injected dropwise, and the solution was refluxed for another 10 min under vigorous stirring. During this process, a gradual color change from yellow to bluish gray and then to light pink of the solution could be observed. The obtained citrate-stabilized Au seeds could be well suspended in water. Then, the Au seeds were grown up to a larger particle size with high SERS activity using the following scheme. When the Au seeds solution was cooled to 90 °C, 1 mL of sodium citrate aqueous solution (60 mM) was injected into it, and 1 mL of HAuCl_4_ (25 mM) solution was injected into the solution after about 30 s. The solution was heated for 30 min under reflux condition. Then, this procedure was repeated twice, three times, and four times (sequential addition of 1 mL of 60 mM sodium citrate and 1 mL of 25 mM HAuCl_4_) to grow up to 2, 3, and 4 generations of gold particles of progressively increasing sizes. During the first growth process, it can be observed that the gold colloid solution gradually changed from light pink to wine red. In the subsequent growth process, the wine red gradually deepened. When reaction was finished, the colloid solution was immediately cooled in the ice-water bath to prevent the nanoparticles from overmaturing. Finally, the obtained AuNPs colloid with different sizes were stored in dark at 4 °C for further experiments.

### 3.4. Preparation of Halide-Modified AuNPs Substrates

The AuNPs were firstly concentrated by centrifugation (6000 r/min, 10 min) to achieve about 2× concentrated AuNPs colloid (by concentrating 2 mL of AuNPs colloid solution to 1 mL). Then, the concentrated AuNPs colloid was mixed with the halide solutions (NaF, NaCl, NaBr, or NaI) at different concentrations (1 × 10^−1^ to 1 × 10^−4^ mol/L) in a 1:1 ratio, and the mixture was allowed to stand at room temperature for 4 min to ensure that the surface of AuNPs was completely modified by halide. No obvious color change was observed after the surface modification of AuNPs. Finally, the halide-modified AuNPs substrate was obtained.

### 3.5. Pretreatment of Wheat Samples

Wheat samples were homogenized using a pulverizer and spiked with the flumetsulam standard solution in different concentration levels (0, 0.05, 0.1, 0.5, 1, 2, 5, 10, 20, 50, and 100 µg/g). Flumetsulam was extracted from the spiked wheat samples using the following pretreatment method. The wheat powder was first filtered through 10-mesh sieves. A total of 0.5 g of wheat powder was mixed with 5 mL of hexane and then incubated for 2 min. Then, the mixture was ultrasonically shaken for 8 min and centrifuged at 10,000 rpm for 5 min. Discard the supernatant and save the residue. Then, 3 mL of acetone was added to the residue, and the mixture was ultrasonically shaken for 10 min and centrifuged at 4000 rpm for 5 min. Then, we moved the supernatant to a concentrated bottle. Wheat residue was extracted using 2 mL of acetone again, and the supernatant was merged with the previous supernatant. All the obtained supernatant was evaporated to dryness under a stream of nitrogen and then redissolved to 200 μL with deionized water. The resulting solution was filtered with 0.22 µm organic filtration, and it was used for SERS measurement directly.

### 3.6. SERS Measurement

SERS samples were prepared for the qualitative identification and quantitative analysis of flumetsulam. First, 30 μL of halide-modified AuNPs colloid solution was added into a 0.6 mL centrifuge tube, and then 30 μL of the flumetsulam standard solution or wheat extract was added into the centrifuge tube. Subsequently, 10 μL of sulfate solution was added in one shot, and the final mixture solution was incubated at room temperature for about 10 s. The color of the resulting mixture quickly turned from wine red to dark blue. Then, it was injected into a 1 cm quartz cell for SERS measurement immediately. The Raman spectra were recorded using the 785 nm laser with 30% power, and the typical collecting time was 2 s with one time accumulation. Considering the complexity of the wheat extract, the collecting time was extended to 20 s, and the power was increased to 40% (≈156 mW).

### 3.7. DFT Calculation

The Gaussian 03 software was used to optimize the geometry and calculate the Raman spectra of flumetsulam in this work. The molecular structure of flumetsulam was optimized using the B3LYP function with a 6-31G* basis set [54,55], and the theory Raman spectrum was further obtained.

### 3.8. Spectral Data Analysis

All Raman spectra were collected using the BWSpec software (version 4.11-1) and saved as a txt file for subsequent analysis. Before establishing the calibration curve, the corresponding SERS spectra were processed with the Savitzky–Golay function in BWSpec software to obtain the second derivative transformation, which can enhance the spectral resolution and adjust the baseline shift [27,56,57]. The other raw spectra were not further processed unless otherwise stated. All spectra in this study were drawn using Origin 8.5 software.

## 4. Conclusions

In summary, the two-step modified concentrated AuNPs substrate was successfully fabricated to detect flumetsulam residue in wheat quickly. Here, the NaCl was served as an activator to modify and activate the AuNPs substrate, so as to detect flumetsulam residue more efficiently. The MgSO_4_ acted as an aggregating agent to form more “hot spots”, thereby achieving more sensitive detection of flumetsulam residue. A linear range from 5 to 1000 μg/L and an LOD of about 1.92 μg/L could be achieved for the detection of flumetsulam standard solution. This SERS method was also applied in the detection of flumetsulam in real wheat samples successfully. With a simple pretreatment, the LOD of flumetsulam residue in wheat could be obtained down to 0.01 μg/g, which matches with the national maximum residue limit of flumetsulam in wheat set in China. The recovery of flumetsulam residue in wheat ranges from 88.3% to 95.6% with RSDs between 5.6% and 8.9%. All these results indicate that the combination of SERS technique and the modified AuNPs can achieve fast, low-cost, and on-site detection of flumetsulam residue in agri-foods fields.

## Figures and Tables

**Table 1:** Recovery test of flumetsulam in real wheat samples by the proposed SERS method (n = 3).

Samples	Flumetsulam Content (μg/g)		Recovery (%)	RSD (%)
	Spiked	Measured		
Wheat	5	4.4137	88.3	8.9
	0.5	0.4782	95.6	5.6
	0.05	0.0445	89.0	7.2

## Supplementary Materials

The following are available online, Figure S1: The SEM image of NaCl-modified AuNPs with flumetsulam (without addition of MgSO_4_); Figure S2: The statistics of particle size distribution of AuNPs; Figure S3: The peak intensity at 786 cm^−1^ of flumetsulam adsorbed on different concentrations (10^−1^ to 10^−4^ mol/L) of NaF, NaCl and NaBr modified AuNPs substrates; Figure S4: The second derivative transformation of SERS spectra of flumetsulam; Table S1: The Raman shifts and band assignments of flumetsulam molecule.
