# Supplementary material for: Fast and Low-Cost Surface-Enhanced Raman Scattering (SERS) Method for On-Site Detection of Flumetsulam in Wheat
Source: Molecules. 2020 Oct 13;25(20):4662. doi: 10.3390/molecules25204662 (PMC7587348; doi:10.3390/molecules25204662)
Supplement: Supplementary file 1 [file molecules-25-04662-s001.pdf]

Supporting information

Fast and Low-cost Surface-enhanced Raman Scattering (SERS) Method for On-site Detection of Flumetsulam in Wheat

Mingming Han, Hongmei Lu, Zhimin Zhang*

College of Chemistry and Chemical Engineering, Central South University, Changsha 410083, P.R. of China; mingminghan@csu.edu.cn (M. H); hongmeilu@csu.edu.cn (H. L.); zmzhang@csu.edu.cn (Z. Z)

* Correspondence: zmzhang@csu.edu.cn; Tel.: +86-13973110646 (Z.Z.)

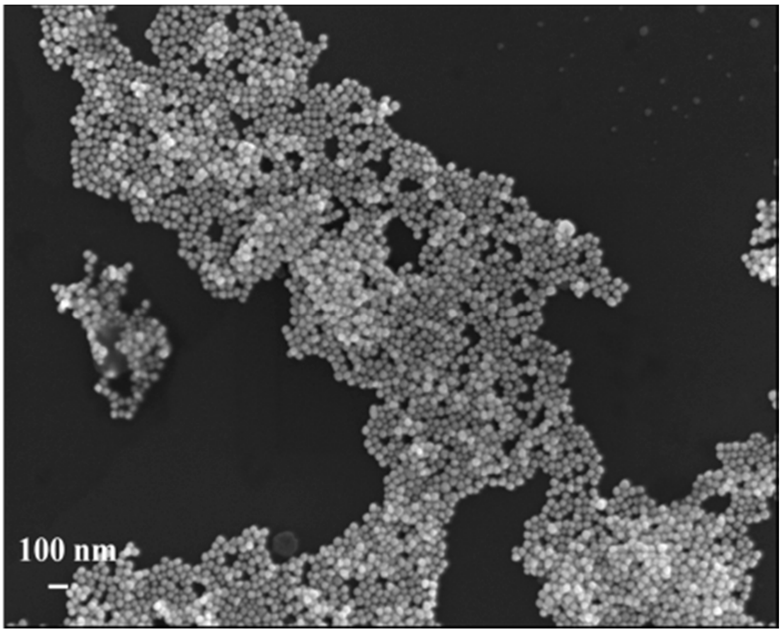

Figure S1. The SEM image of NaCl modified AuNPs with flumetsulam (without addition of $MgSO_4$).

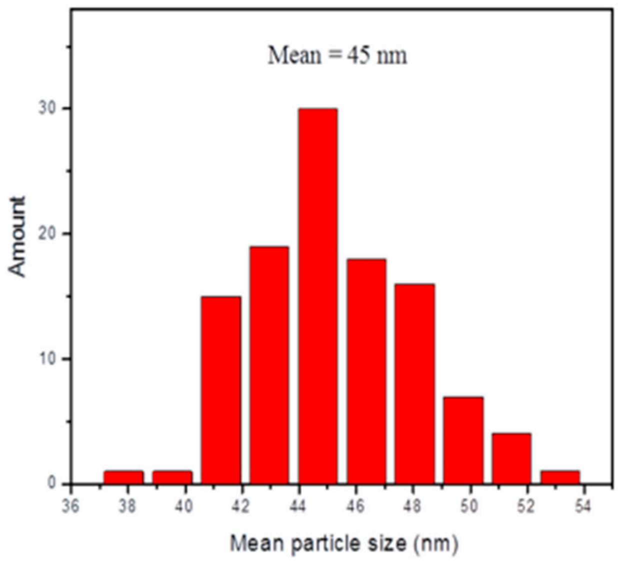

Figure S2. The statistics of particle size distribution of AuNPs.

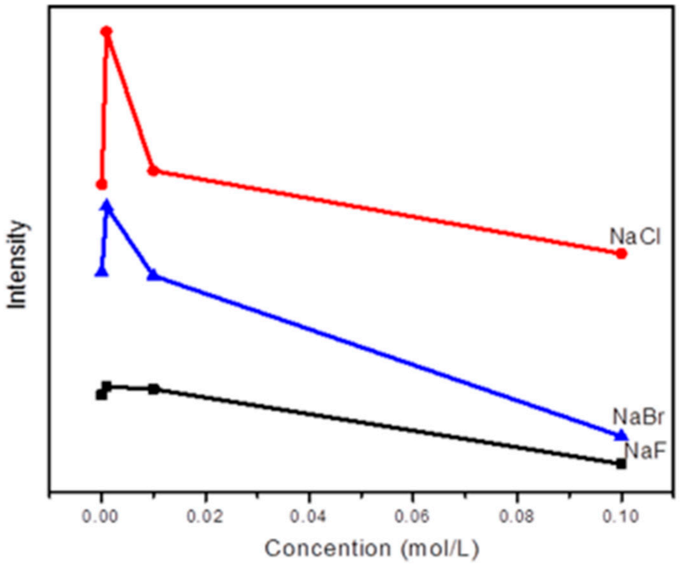

Figure S3. The peak intensity at 786 cm^{-1} of flumetsulam adsorbed on different concentrations (10^{-1}~10^{-4} mol/L) of NaF, NaCl and NaBr modified AuNPs substrates.

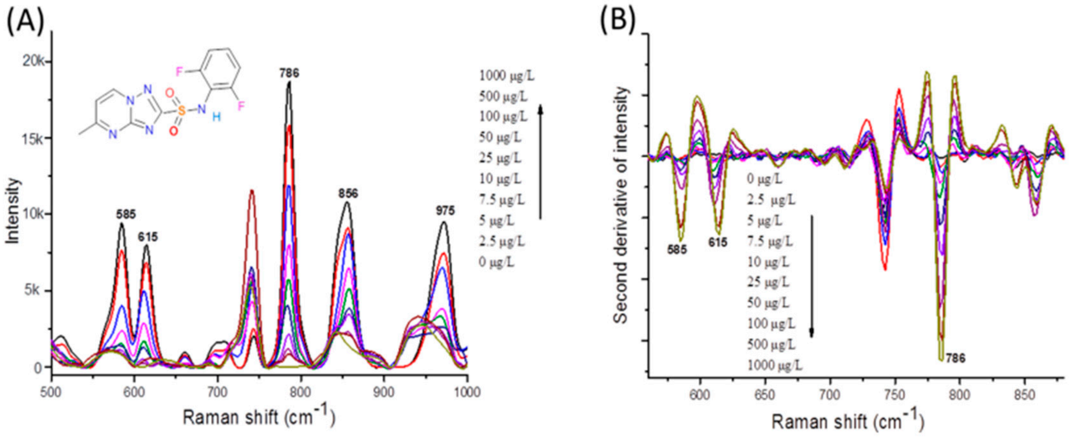

Figure S4. (A): SERS spectra of flumetsulam standard solution with different concentrations absorbed on the two-step modified concentrated AuNPs substrate. (**B**): The second derivative transformation of SERS spectra of flumetsulam in the range of 580~850 cm^{-1}.

Table 1. The Raman shifts and band assignments of flumetsulam molecule.

Flumetsulam	Raman (cm^{-1})	SERS (cm^{-1})	Vibrational modes
		585	β(ring A); β(ring B); γ(-SO_2)
		615	β(ring A); ν(C_3-S)
	776	786	β(ring A); ν(N-S); ν(C_7-C_{10}); β(ring C)
	844	856	β(ring A); β(ring B); ν(C_3-S)
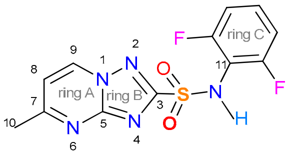	992	975	change of angle $N_2C_3N_{4(\text{ring B})}$; δ(-$C_{10}H_3$); ν($C_3$-S)
	1060	1064	β(-CH)$_{\text{ringC}}$
	1622	1614	β(ring C); ν(C_{11}-N)

i: ν-stretching vibrations; β -bending in-plane; γ -bending out-of-plane; δ -deformation.
